# Psychometric properties of ViewMind Atlas™: a Digital Cognitive Biomarker for Alzheimer's disease

**DOI:** 10.1002/alz70856_106647

**Published:** 2026-01-08

**Authors:** Mario A A Parra, María Bárbara Eizaguirre, Ricardo N Alonso, Danilo Verge, Gerardo Fernandez

**Affiliations:** ^1^ University of Strathcylde, Glasgow, Glasgow, United Kingdom; ^2^ Ramos Mejía Hospital, Buenos Aires, Buenos Aires, Argentina; ^3^ ViewMind, NY, NY, USA

## Abstract

**Background:**

ViewMind Atlas™ detects patients with or at risk of Alzheimer's disease (AD) with high precision (Fernandez et al., 2022). It incorporates a head‐mounted display (HMD), which measures eye‐tracking behaviors during five validated visual tasks. The biomarker identifies carriers of autosomal dominant mutations in preclinical and prodromal stages and prospectively predicts, with over 96% accuracy, sporadic MCI to AD conversion within a 3‐year window (Parra et al., 2022). ViewMind Atlas™ correlates with blood‐based biomarkers linked to inflammatory mechanisms in preclinical AD (Parra et al. (2024). We investigated the psychometric properties of this promising neurocognitive biomarker in a prospective clinical study (clinicaltrials.gov, ID NCT06746844).

**Methods:**

Participants with and without cognitive complaints (*n* = 141) were assessed with ViewMind Atlas™, MoCA, and neuropsychological tests. We explored test‐retest reliability using Kendall's coefficient of concordance (W) (*n* = 27, Age: 59.7±9.2, Education 14.7±4, 22 female), construct validity using Pearson correlations (whole sample aged 45–95 years ), and Discriminability between self (*n* = 48) vs clinic referrals (*n* = 41) (Age: 66.2±10, Education 13±7.7, 65 female) using t‐tests.

**Results:**

All ViewMind Atlas™'s domains (Working Memory, Perception, Processing Speed, Sustained Attention, Selective Attention, Executive Function, and the overall Composite) showed high test‐retest agreement (W range: 0.82‐0.89, all *p* < 0.05). ViewMind metrics significantly correlated with neuropsychological tests of equivalent constructs (Executive Function vs. MoCA Executive, r=0.329, *p* <0.01, Sustained Attention vs. MoCA Attention, r=0.354, *p* <0.01; Processing Speed vs. DSST, r=0.548, *p* <0.01, Working Memory vs. ROFC, r=0.3836, *p* <0.01). Self‐referrals presented with a more pronounced amnesic and dysexecutive profile than clinic‐referrals, as informed by ViewMind Atlas™ and traditional assessments.

**Conclusion:**

ViewMind Atlas™ is psychometrically valid. Its test‐retest reliability confirms its usefulness for serial testing. Its convergent validity relative to traditional neuropsychological tests endorses the proposed constructs and the theory‐driven nature of this novel neurocognitive biomarker. ViewMind Atlas™ discriminability (self vs clinic referrals) confirms its clinical utility in assessing older adults worried about their brain health who seek healthcare.

**References**:

1. Fernandez et al. (2022). Alzheimers Dement, doi: 10.1002/alz.066784.

2. Parra et al. (2022). Alzheimers Dement (Amst). doi: 10.1002/dad2.12386

3. Parra et al. (2024). JPAD, 17^th^ CTAD Proceedings, 12, 100061 (LP052), pp. 103.

